# Patient-centered care in the emergency department: a systematic review and meta-ethnographic synthesis

**DOI:** 10.1186/s12245-022-00438-0

**Published:** 2022-08-11

**Authors:** Anna Walsh, Elnaz Bodaghkhani, Holly Etchegary, Lindsay Alcock, Christopher Patey, Dorothy Senior, Shabnam Asghari

**Affiliations:** 1 Centre for Rural Health Studies, Discipline of Family Medicine, Faculty of Medicine, Memorial University of Newfoundland and Labrador, St. John’s, Newfoundland and Labrador Canada; 2 Emergency Medicine Department, The University of British Columbia, Vancouver, Canada; 3 Health Sciences Library, Faculty of Medicine, Memorial University of Newfoundland and Labrador, St. John’s, Newfoundland and Labrador Canada; 4 Discipline of Family Medicine, Faculty of Medicine, Memorial University of Newfoundland and Labrador, St. John’s, Newfoundland and Labrador Canada; 5 Community Scholar with Center for Rural Health Studies, Discipline of Family Medicine, Memorial University of Newfoundland, St. John’s, Newfoundland and Labrador Canada; 6 Department of Family Medicine, Centre for Rural Health Studies Faculty of Medicine, Memorial University of Newfoundland and Labrador Health Sciences Centre, 300 Prince Philip Drive, NL A1B 3V6 St. John’s, Canada

**Keywords:** Emergency department, Patient-centered care, Patient engagement

## Abstract

**Background:**

Patient-centered care (PCC) is an emerging priority in many healthcare settings but lacks clarity in the emergency department (ED). It is of interest to know what PCC practices are most important to patients to better their experience. The objective of this study was to conduct a mixed-methods systematic review of PCC in the ED.

**Methods:**

We used stakeholder and patient engagement to consult with clinicians, subject-matter experts, patient partners, and community organizations to determine patient needs. We examined all articles in the ED context with PCC as the intervention. Two independent reviewers screened 3136 articles and 13 were included. A meta-ethnographic analysis was conducted to determine common themes of PCC.

**Results:**

Themes included emotional support, communication, education, involvement of patient/family in information sharing and decision making, comfort of environment, respect and trust, continuity, and transition of care. Challenges in the ED reflected a lack of PCC. Moreover, implementation of PCC had many benefits including higher patient satisfaction with their care. Though there were commonalities of PCC components, there was no consistently used definition for PCC in the ED.

**Conclusion:**

The findings of this review support the evidence that PCC is of high value to the ED setting and should be standardized in practice.

**Supplementary Information:**

The online version contains supplementary material available at 10.1186/s12245-022-00438-0.

## Background

Patient-centered care (PCC) is a method of forming trusting relationships between patients and care providers. It is widely defined as a holistic approach to providing care that includes patient involvement, communication, access to services, well-trained staff, and an environment that meets patients’ psychosocial, physical, and cultural needs [1]. It has previously been explored in many fields of healthcare including, but not limited to nursing [2], cancer care [3], pediatrics [4], long-term care [5], mental health [6], primary care [7], and related areas such as social work [8]. PCC requires efforts on all levels including the patient, the provider, and the healthcare system [9] to ensure it is meaningfully practiced.

Effective PCC should help patients and physicians to communicate in a respectful way that both parties understand within an environment that is conducive to appropriate care processes. Previous research demonstrates that when there is dissonance between patients’ expectations and the services rendered, there are often components of patient-centeredness missing [10–12]. PCC and its many components can make a huge impact on patients’ experiences when performed properly.

PCC is an emerging priority in many healthcare settings, yet it has not been incorporated into ED practice in a standard way. Traditional ED quality improvement initiatives often focus on structures, processes, and outcomes—for example, how long a patient waits, the percentage of patients that leave without being seen by a physician, and the volume of patients during the intervention [13, 14]. Although these variables should be considered to create a better ED that benefits the health system, the way in which patients perceive their experience is essential to acknowledge. The quality and personalization of services sought out by patients in the ED are critical, and it is highly important to ensure patients leave feeling satisfied with the care they receive. Despite the growing literature on interventions that can be used to make the ED more efficient [15–18], there are currently no systematic reviews on how EDs include PCC. Thus, it is of interest to know what is most important to patients to better their experience, and how PCC can encompass those elements.

The goal of this review is to examine PCC in the ED to better understand how EDs undertake this method. The objectives are to determine (1) what the components of PCC are in the ED and (2) what the challenges and benefits of PCC in the ED are, as perceived by staff and patients.

## Methodology

This study follows the strategy of review outlined in the protocol available on PROSPERO, updated in November 2021 [19]. As stated in the protocol, our phenomenon of interest included PCC in the context of the ED, and our main outcomes of interest are components or methods of PCC, challenges and benefits to PCC for staff, and challenges and benefits to PCC for patients. Secondary outcomes of interest included any evidence on the impacts of PCC, e.g., ED wait times or length of stay, patient satisfaction, and patients leaving without being seen.

### Patient and public involvement

**Fig. 1:**
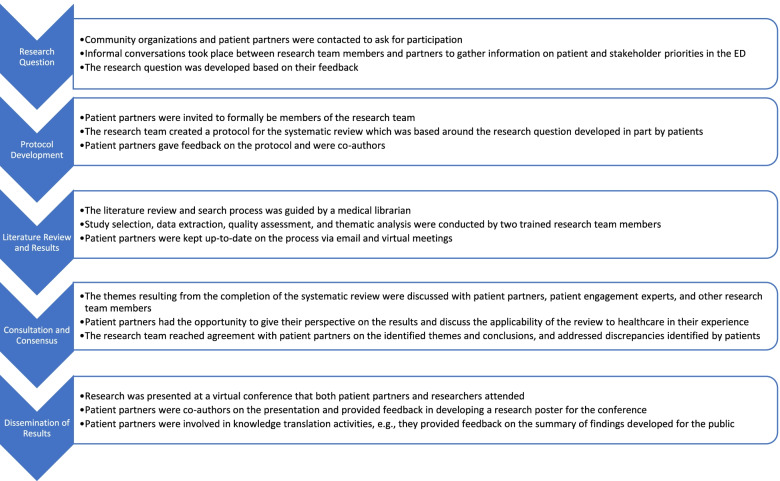
Pathway of patient and public involvement in the study

To better understand current and previous experiences of patients in the ED, we undertook patient engagement initiatives [20]. By involving patient partners, the research in question becomes more patient-centered [21]. We discussed issues and needs with local advocacy groups to gain perspective from those with lived experience and included patient partners on the research team. Figure 1 depicts the process of patient involvement throughout the study from research question development to dissemination.

### Search strategy

Search terms were identified and search strategies were developed by a medical librarian. The primary strategy (PubMed, MEDLINE) was peer-reviewed using PRESS and translated to search Embase (Elsevier), CINAHLPlus (EBSCO), PsycINFO (EBSCO), and Cochrane (Wiley) (Additional file 1: Appendix A). The reference sections of relevant studies were also examined for any additional references. The original searches were completed on June 6, 2020, and rerun on December 2, 2020.

### Study selection

The titles and abstracts of all articles identified by the database searches were examined by two independent reviewers after duplicates were removed. Inclusion and exclusion criteria can be found in the protocol [19]. Reviewers completed a calibration exercise with the first ten articles and reached agreement on inclusion criteria. All full texts were then reviewed. A third reviewer was invited to mitigate any dispute.

### Quality appraisal

The quality of each study was assessed using the scoring system for mixed studies reviews [22]. Studies 75% and above were good quality, 50–74% were fair, and below 50% were low. All studies that met the criteria were included, despite the quality score.

### Synthesis of results and analysis

Two approaches were used for data synthesis and integration. The Joanna Briggs Institute (JBI) convergence-integrated approach for mixed-methods studies [23] was used to “qualitize” quantitative studies into textual descriptions to allow integration with qualitative data. “Qualitized” findings from quantitative studies are assembled into categories with qualitative findings based on similarity of meaning. To best determine the categories once all data was “qualitized”, we used a meta-ethnographic approach. Meta-ethnographic synthesis is suitable for understanding conceptual or theoretical underpinnings of a particular phenomenon [24]. This approach was selected to help understand what the various components of PCC were and what common components were used across all included studies. It was also used to compile information regarding challenges and benefits of PCC and determine commonalities across the literature. For the purposes of this study, “concepts” are defined as information extracted from the studies that include either direct quotes from study participants or authors’ interpretations of their own results. “Key concepts” are the groupings of similarities and differences across concepts from the included studies after the studies are translated into one another, and “themes” are the third-order constructs that are re-interpretations of the concepts and key concepts determined by the reviewers of this study.

The concepts were separated based on the viewpoint, being staff or patient/family. To address each population, healthcare providers, and patients and families, two separate reciprocal translations were conducted. All concepts were compared to one another using a line of argument synthesis [24] to identify key concepts reflected in both populations that described PCC activities, challenges, and benefits. Theme interpretation was completed by one reviewer (AW), based on the independent data extractions from both reviewers. All themes were discussed during weekly meetings between members of the research team, including a PCC expert and clinician, patient engagement expert, and methodologist, to reduce bias and ensure consensus was reached. Finally, the synthesis is expressed through tables and narrative format [25].

## Results

### Study characteristics

**Fig. 2:**
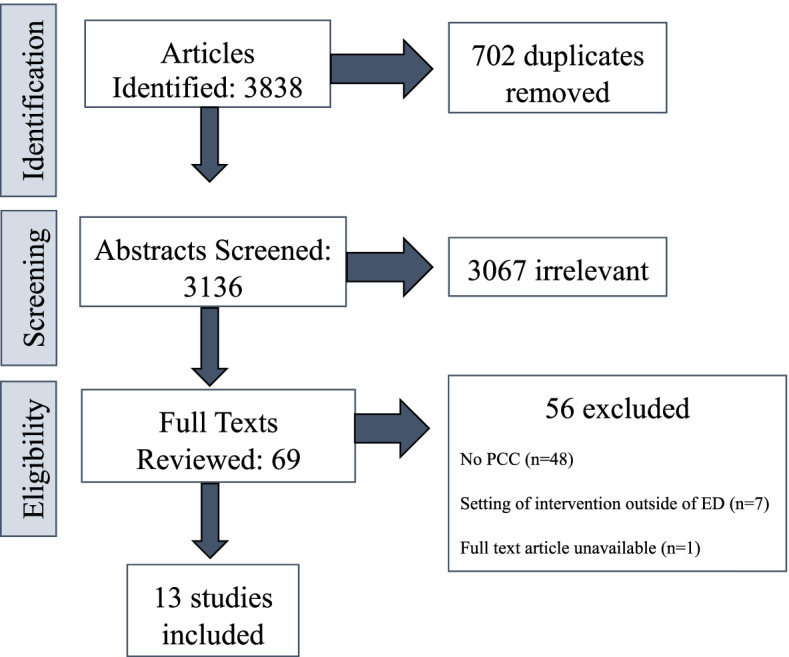
PRISMA diagram

Three thousand eight hundred thirty-eight studies were imported for screening. 3136 total articles were screened in the title and abstract phase, 69 were assessed for eligibility in the full-text phase, and 14 studies were included in the data extraction phase. Reasons for exclusion were no patient-centered care (*n*=48), setting of intervention outside of the ED (*n*=7), and full-text article was not available (*n*=1). One study was removed during the extraction phase because the focus of the article was social services rather than healthcare, despite being in the ED setting, leaving 13 articles for the final data extraction and quality appraisal (Fig. 2).

Table 1 demonstrates all study characteristics. Countries of study included the USA (*n*=4), Canada (*n*=2), UK (*n*=2), Sweden (*n*=2), Australia (*n*=2), and Taiwan (*n*=1). All studies included patient and/or healthcare professional populations that had experience in the ED. Eleven studies included populations related to adult ED care and two studies included populations related to pediatric ED care. While most studies were directly related to the ED setting (*n*=10), three studies investigated settings that were specialized units adjoined to or having direct impacts on EDs (*n*=3). The quality scores varied. Six studies (*n*=6) were considered good quality, six (*n*=6) were fair, and one (*n*=1) low.

**Table 1:** Characteristics of included studies

Author (year)	Country	Study objective	Study design	Study population	Sample size	Quality score
Nicholas (2020) [26]	Canada	To determine how patient and family-centered care is demonstrated in the ED for children with autism	Semi-structured interviews	Parents and family members of children with autism spectrum disorder, from two large Canadian EDs; ED-based health care providers	53 (10 physicians, 12 nurses, 31 parents of patients)	75%
Innes (2018) [27]	Australia	To identify the activities and behaviors, including patient-centered care behaviours, of waiting room nurses (WRN) in the ED	Observational study	WRN from two EDs	8 WRN observed across 13 sessions	66.6%
Polevoi (2013) [28]	USA	To compare a traditional resident consultation model with a co-management model, where the psychiatric consultation and liaison service assumes full responsibility for providing direct psychiatric care, to reduce length of stay for patients with psychiatric emergencies in the ED	Prospective cohort study	Patients in a 29-bed urban academic ED between 2007 and 2009	1884 patient visits	66.6%
Frank (2009 B) [29]	Sweden	Interviews to describe patients’ perceptions of patient participation in their care in the ED	Phenomenographic study	Patients who attended one metropolitan ED in 2006	9 patients interviewed	66.6%
Frank (2009 A) [30]	Sweden	Interviews to describe care giver’s perceptions of patient participation in their care in the ED	Phenomenographic study	Health care providers in one metropolitan ED	11 health care providers (4 registered nurses, 4 auxiliary nurses, 3 physicians)	75%
Steven (2015) [31]	USA	To determine how the CARES unit, a crisis stabilization unit, influences length of stay and costs for psychiatric patients in the pediatric ED	Retrospective, chart review	All psychiatric patients presenting to the ED of a children’s hospital between 2006 and 2008	1719 patients pre-CARES and 1863 patients post-CARES	66.6%
Wilhelm (2007) [32]	Australia	To examine the effectiveness of the clinic (a novel brief intervention service for patients presenting to the emergency department following deliberate self harm—DSH—or with suicidal ideation) in terms of service utilization and patient and clinician feedback and to explore the correlates of repeated DSH	Retrospective, chart review	Patients with deliberate self-harm or suicidal ideation who presented at an emergency department and got a “green card,” a referral to the clinic	456 patients were referred to the clinic	33.3%
Walker (2016) [33]	UK	To explore nursing interventions for person-centered bereavement care in adult acute and critical care settings; Provide insight into nurses’ experiences of care for the suddenly bereaved; Examine the provision of person-centred bereavement care; Inform the development of person-centeredness in practice	Descriptive exploratory study	Nurses who work in hospital areas where patient death is common and they participate in bereavement care, e.g., emergency/acute care, critical care, cardiac care	10 (4 emergency care nurses, 4 critical care nurses, 2 cardiac care nurses)	91.6%
Trethewey (2019) [34]	UK	To evaluate the activities of the psychiatric decisions unit (PDU) and its impact on the frequency of ED presentations and inpatient admissions, and patient satisfaction with the PDU services	Quasi-experimental study	Psychiatric patients referred to the PDU either by a street triage team or a rapid assessment interface and discharge team	385 patients referred to the PDU; 80 patients provided feedback on satisfaction with the PDU	50%
Zeller (2014) [35]	USA	To assess the effects of a regional dedicated emergency psychiatric facility design—the Alameda Model—on boarding times and hospitalization rates for psychiatric patients in the ED	Cross-sectional	Patients who presented to the ED on an involuntary mental health hold	144 patients	83.3%
Wang (2016) [36]	Taiwan	To explore the medical decision-making process of patients in the ED	Grounded theory, interviews	Patients of an ED between June-December 2011	30 patients interviewed	75%
Heifetz (2018) [37]	Canada	To evaluate communication tools to be used by people with intellectual and developmental disabilities (IDD) in psychiatric and general EDs in three regions of Ontario, Canada	Descriptive study, evaluation	Stakeholders (e.g., hospital staff, community agency representatives, families) and individuals with IDD	18 stakeholders completed interviews 28 caregivers, parents, and individuals with IDD completed feedback questionnaires	58.3%
Owens (2007) [38]	USA	To define exposure to intimate partner violence (IPV) among adult female patients in emergency psychiatric settings and the relationship between the dependent variable of disclosure of IPV in a psychiatric emergency setting to clinical staff and the independent variable of perception of the provider by respondents	Descriptive, exploratory	Adult women who present for emergency psychiatric evaluation who have experienced a form of IPV	216 patients	75%

### Defining components of patient-centered care

Descriptions and definitions of PCC were extracted from all included articles (Table 2). Five articles had directly stated definitions or descriptions of patient-centered, person-centered, and family-centered care. The other nine articles did not describe their activities using PCC-related terminology but were still included due to the presence of PCC components. The most cited components of PCC in the literature, and thus, the themes of PCC, were comfort of environment (*n*=8), communication (*n*=7), education (*n*=7), involvement of patient/family in information sharing and decision making (*n*=7), respect and trust (*n*=7), continuity and transition of care (*n*=7), and emotional support (*n*=5) (Table 3).

**Table 2:** Definitions of PCC and key findings as identified by the included articles

Author (year)	Description of PCC	Comparison group	Key finding
Nicholas (2020) [26]	(1) Dignity and respect (listening to families and incorporating their values, knowledge and beliefs in care), (2) participation (families are encouraged to participate in care and decision-making to the level they choose), (3) collaboration (families are involved in care delivery, institutional policy and program development, and (4) information sharing (timely, complete and accurate information shared with families).	No comparison	Patient and family-centered care in the ED for children with autism spectrum disorder is strongly recommended and beneficial for patients, family, and staff.
Innes (2018) [27]	Patient-centered care was given to patients by being respectful, empathetic, and sincere when listening to patient histories; ensured that patients and families were involved in all discussions and decisions relating to their presentation and plan; clarify unclear points and use language/terminology appropriate for specific patients.	No comparison	Therapeutic engagement of emergency nurses with patients in the waiting room allowed them to deliver patient-centered, holistic, supportive, and informative care.
Polevoi (2013) [28]	Patient-centered care was reflected in the coordination of services (continuity of care) provided to patients in the ED, and access to psychiatric services in the ED through staff collaboration.	Traditional resident consultation model compared to new co-management model	The co-management model marked a reduced length of stay for all psychiatric patients and a decrease in the number of patients who left without being seen.
Frank (2009 B) [29]	Patient participation is a component of patient-centered care. Patient participation was defined by authors as having the right and duty to participate individually and collectively in the planning and implementation of their health care; it requires that formal health carers are willing to focus on the interpersonal relationship between patients and carer, as well as having an attitude that enables patients to relate to them as subjects taking part in care	No comparison	Patients go through different stages of participation in care and have different needs for participation. This has important implications for ED staff in practice.
Frank (2009 A) [30]	Patient participation is a component of patient-centered care. Patient participation was defined by authors as having the right and duty to participate individually and collectively in the planning and implementation of their health care; it requires that formal health carers are willing to focus on the interpersonal relationship between patients and carer, as well as having an attitude that enables patients to relate to them as subjects taking part in care.	No comparison	Patient participation is conditional of the healthcare providers or caregivers. It is most often circumstantial, and difficult for caregivers when dealing with aggravated patients.
Steven (2015) [31]	The CARES unit reflects collaboration of ED and psychiatric staff, accommodating parents and guardians to remain with children moved to the unit, and rapid stabilization via access to resources in an appropriate environment.	Pre-intervention group compared to post-intervention group	The length of stay in the ED after the implementation of CARES was significantly reduced, as was the ED cost per patient.
Wilhelm (2007) [32]	The intervention’s patient-centered component involved providing patients with a choice of problem area, i.e., what they wanted to work on most for themselves, and that that change was empowering for them while also providing a “taste” of what psychotherapeutic interventions have to offer. This reflects patient involvement in their own care and decision making.	Comparisons were made between repeat and first-time patient groups	The green card clinic provides a patient-centered, collaborative approach to intervention following self-harm and continuity of care through increased attendance in follow-up sessions.
Walker (2016) [33]	Person-centered care places patients and families at the heart of care decisions. The person-centered nursing framework was used to code material based on five care processes: working with patient’s beliefs and values, engagement, having sympathetic presence, sharing decision-making, and providing holistic care.	No comparison	Establishing a team philosophy of person-centered care can help promote consistency in the experiences of suddenly bereaved families.
Trethewey (2019) [34]	Respect and understanding are components of patient-centered care that was measured using the patient satisfaction and feedback forms in this study. Patients felt they were treated with respect and understanding	Pre-PDU data was compared to post-PDU data	The PDU helps to relieve psychiatric pressure on busy EDs and creates a more optimum environment for psychiatric assessment.
Zeller (2014) [35]	The Alameda model involves healthcare provider collaboration across EDs and EMS services to transfer patients to appropriate EDs equipped to handle psychiatric emergencies.	Earlier data collected in a 2012 California hospital survey	The Alameda model boarded psychiatric patients in the ED for 80% less time.
Wang (2016) [36]	A component of patient-centered care is sharing in decision making processes with the patient. This study breaks the decision-making process down into three phases and identifies how the patient and provider can problem-solve and make healthcare decisions collaboratively.	No comparison	Decision making processes occur in different stages and ED staff should support patients faced with complex medical decision making through advocacy, patient-centered care, shared decision-making, and education.
Heifetz (2018) [37]	Person-centered approach by easing communication between providers and patients with IDD	No comparison	The health passport tool allows patients to more easily communicate with healthcare providers but continued efforts are needed to educate staff on how to look for and use the tool effectively.
Owens (2007) [38]	Patient-centered behaviors of providers include measures of trust, interpersonal interactions, communication, and knowledge of the patient as a person.	Group of women who disclosed their history with IPV to their physician was compared to a group that did not disclose their history with IPV in terms of patient-centered behaviors demonstrated by the physician	Patient-centered behaviors play a role in assisting female abuse victims to disclose their experience with IPV. The perception of a provider as knowledgeable about their patients was associated with increased disclosure.

**Table 3:** Components of PCC as identified within the included articles

	Communication	Education	Involvement of patient/family in information sharing and decision making	Comfort of environment	Respect and trust	Emotional support	Continuity and transition of care
Nicholas (2020) [26]	**X**	**X**	**X**	**X**	**X**	**X**	**X**
Innes (2018) [27]	**X**	**X**	**X**	**X**	**X**	**X**	**X**
Polevoi (2013) [28]				**X**			**X**
Frank (2009B) [29]	**X**		**X**	**X**	**X**	**X**	
Frank (2009A) [30]	**X**		**X**				
Wilhelm (2007) [32]		**X**					**X**
Walker (2016) [33]	**X**	**X**	**X**	**X**	**X**	**X**	
Trethewey (2019) [34]				**X**	**X**		**X**
Zeller (2014) [35]				**X**			**X**
Wang (2016) [36]	**X**		**X**		**X**		
Heifetz (2018) [37]	**X**	**X**	**X**	**X**			
Owens (2007) [34]		**X**			**X**	**X**	
Steven (2015) [31]							**X**

### Challenges and benefits of PCC as perceived by ED staff

Challenges and benefits of providing PCC were noted in four articles (*n*=4). Noted concepts of difficulties in providing components of PCC from the ED staff perspective were a lack of training or experience (*n*=2), communication barriers by having multiple care providers (*n*=1), complex patient needs (*n*=1), the design of the ED space being set up for efficiency rather than communication (*n*=2), patient frustration and negative attitudes (*n*=1), work demands impacting providers’ ability to form relationships with patients and families (*n*=1), and professional conflicts impacting trust between patients and providers (*n*=1). However, components of PCC that were applied successfully saw beneficial results. ED staff reported that keeping patients informed helps to avoid emotional distress and uncertainty (*n*=2), patient placement in close proximity to ED staff with clear lines of sight allows staff to offer their presence (*n*=1), and encouraging patient participation (*n*=1) and treating patients and families as experts in their own care (*n*=1) brings about patient-provider collaboration.

### Challenges and benefits of PCC as perceived by patients

Patient experiences were described in six articles (*n*=6) demonstrating evidence of barriers to and benefits of receiving PCC. Concepts and key concepts of patient concerns included overwhelming waiting rooms (*n*=1), difficulty of navigation (*n*=1), and untrained or inexperienced staff (*n*=2). Further barriers to a positive care experience were lack of frequent updates from staff (*n*=1), limited access to information on ED care processes (*n*=1), dismissive attitudes from ED staff towards patients’ and families’ input (*n*=2), difficulty establishing communication with ED staff (*n*=1), and the use of language by staff that patients cannot understand thus limiting their ability to participate in decision making (*n*=1). Patient satisfaction was often achieved when components of PCC were present, for example, having frequent contact with staff (*n*=1), when patients felt listened to and valued as experts in their own health (*n*=2), when interacting with trained staff (*n*=1), when respected by ED staff (*n*=2), and being treated courteously without scepticism (*n*=1). When they were able to establish relationships, patients were able to share more information with providers (*n*=1) and place their trust in care providers to make good medical decisions on their behalf (*n*=1). Furthermore, having a patient-focused environment with accessible features allows patients to be comfortable in the ED (*n*=1) and having continuity of care via follow-up clinics helped patients to make changes to their lifestyles and attitudes (*n*=1) that in turn better their health.

### Impacts of PCC on outcomes

Four studies (*n*=4) assessed the impacts of PCC components on various outcomes in the ED. Examples of quantitative impacts measured included patient length of stay (*n*=3), number of patients who left without being seen (*n*=1), and patient satisfaction (*n*=1). Results of these studies demonstrated decreased length of stay (*n*=3), reduced number of patients who left without being seen (*n*=1), and greater patient satisfaction (*n*=1) with the implementation of PCC-related interventions. Further qualitative findings support the idea that the patient experience is bettered by the presence of PCC components both individually and altogether.

### Contribution of patient engagement

The results of this study were shared with patient partners for feedback. There was an agreement on the components of PCC in the ED that were identified, but it was noted that “building trust between patients and providers” might be another important component to consider. Inclusivity and ethnic representation among physicians were identified by patient partners as foundations to building better relationships as it helps patients feel like they can better relate to their care providers. Although these were not components identified in the literature, this may represent another gap in the knowledge of providing PCC in the ED when trying to meet patients’ cultural and psychosocial needs.

## Discussion

PCC can be a valuable contribution to emergency medicine practices. PCC in the ED includes aspects of communication, education, involvement of the patient/family in information sharing and decision making, comfort of environment, respect and trust, emotional support, continuity, and transition of care. However, there is not yet an operational definition for how PCC should be implemented in the ED. Though all the included studies shared common components, most of the studies did not include each component of PCC that was identified. This finding demonstrates there is no agreed-upon framework for PCC in the ED setting. This is echoed throughout the literature and identifies a concern that there is currently a lack of consistency in PCC models throughout the broader healthcare system. Where some studies lack multiple components of PCC, it is possible that they could have had better outcomes had they included the other aspects.

The PCC in the reviewed articles also varied greatly depending on what roles staff had, patients’ illnesses, and the care process involved, e.g., to move them quickly to a specialized unit for appropriate care or to make them more comfortable in the ED waiting room. The variations in perspective likely contributed to the resulting differences across the studies regarding what PCC was practiced and what patients or staff found to be beneficial or lacking. Additionally, there were many identified challenges echoed from both the staff and patient perspectives. For example, staff education was seen as a barrier and communication as an enabler to PCC by both the patients and the staff across multiple studies. This indicates that there is agreement and that the impact of PCC reverberates both positively and negatively throughout the healthcare system. This overlap may suggest a few key starting points to creating a unique definition for PCC in the ED.

Compared to models of PCC in different healthcare settings, there is an overlap of pillars that support patient-centered practices. In a review of over 900 studies on PCC across various healthcare settings [1], a few of the most common principles included taking a holistic approach, seeing the patient as an expert in their own care, recognizing autonomy and sharing responsibility in decision making, ensuring services are accessible, and having supportive, well-trained staff who can communicate and engage with patients. Further frameworks [39, 40] outline that concepts related to the patient-centered environment include advocacy, values, and empowerment as well as staff being partners in care through collaboration, communication, and health promotion. Although five of the included articles in the current review examined populations with mental health emergencies, we did not identify any new themes through analysis and comparison between mental health and non-mental health populations. This contributes to the evidence that different healthcare settings may put emphasis on the components that are more relevant to their context, but the broad ideas of PCC are aligned with the current findings and support the notion that PCC in the ED does not need major adaptations to be integrated. It should also be recognized that components of PCC were practiced before evolving into what is known as PCC today [41]. Therefore, the conception of new PCC pillars throughout different healthcare settings is to be expected. Although the ED environment presents unique challenges, including patient-centeredness can help to create a better environment for providers and patients.

Until now, the components that should go into PCC in the ED have not all been recognized. Rather, they were accounted for piece by piece and not as a whole. One could argue that providing any one component of PCC is better than none; however, it is important to consider all components in a holistic, well-rounded patient-centered practice.

This review can be useful as a foundation to understanding the components of PCC that will improve the ED experience. It can also be used to assist in the development of PCC training modules for ED staff or implementation of better PCC practices in the ED. By using the outlined components of PCC and implementing some of the suggested methods and examples from the literature, it is possible to develop a comprehensive list of actionable PCC practices.

## Limitations

The results of this review are limited by the evidence from the retrieved studies and by the quality of the information reported in those studies. In the quantitative articles, neither the effect sizes nor significance levels were provided in some cases, so we were unable to report this information. The data also did not support meta-analysis, which would have made our results stronger, due to the lack of quantitative evidence. Articles that are aligned with the current evidence are more likely to get published; therefore, it is possible that evidence opposing the included studies was not available due to publication bias. Our search was limited to only English language, peer-reviewed articles; therefore, it is possible articles in other languages or non-peer-reviewed articles were missed. Finally, because some of the included studies had small sample sizes, it is possible that the evidence from those EDs would not be generalizable to another population. In future, should more quantitative evidence become available, it would be beneficial to do quantitative analysis to get a better understanding of the effect of PCC on patient outcomes. The strength of the evidence produced through this review should also be evaluated as new information becomes available surrounding PCC in the ED.

## Conclusion

Despite the challenges faced by staff, patients, and families, PCC overall has a beneficial impact. Some of the many downfalls of ED care identified by its users can be mitigated by implementing PCC. This study contributes to the literature on how we can address PCC in the ED and how it can be used to improve the ED environment. PCC has been identified by patients as essential to improving the patient experience and should be prioritized as an evidence-based method of providing care that meets the patients’ needs.

## Supplementary information

**Additional file 1: Appendix A.** Sample search strategy.

## Data Availability

Data sharing is not applicable to this article as no datasets were generated or analyzed during the current study.

## References

[1] Helping measure person centred care [Internet]. [cited 2021 May 3]. Available from: http://www.health.org.uk/sites/default/files/HelpingMeasurePersonCentredCare.pdf

[2] McCormack B, McCance TV (2006). Development of a framework for person-centred nursing. J Adv Nurs.

[3] McCormack LA, Treiman K, Rupert D, Williams-Piehota P, Nadler E, Arora NK (2011). Measuring patient-centered communication in cancer care: a literature review and the development of a systematic approach. Soc Sci Med.

[4] O’Malley PJ, Brown K, Krug SE, and the Committee on Pediatric Emergency Medicine (2008). Patient- and family-centered care of children in the emergency department. Pediatrics..

[5] Chaudhury H, Hung L, Badger M (2013). The role of physical environment in supporting person-centered dining in long-term care: a review of the literature. Am J Alzheimers Dis Other Dement.

[6] Martin GW, Younger D (2001). Person-centred care for people with dementia: a quality audit approach. J Psychiatr Ment Health Nurs.

[7] Santana MJ, Manalili K, Jolley RJ, Zelinsky S, Quan H, Lu M (2018). How to practice person-centred care: a conceptual framework. Health Expect.

[8] Chung K (1993). Brief social work intervention in the hospice setting: person-centred work and crisis intervention synthesized and distilled. Palliat Med.

[9] Epstein RM, Street RL (2011). The values and value of patient-centered care. Ann Fam Med.

[10] Plewnia A, Bengel J, Körner M (2016). Patient-centeredness and its impact on patient satisfaction and treatment outcomes in medical rehabilitation. Patient Educ Couns.

[11] Kuipers SJ, Cramm JM, Nieboer AP (2019). The importance of patient-centered care and co-creation of care for satisfaction with care and physical and social well-being of patients with multi-morbidity in the primary care setting. BMC Health Serv Res.

[12] Wolf DM, Lehman L, Quinlin R, Zullo T, Hoffman L (2008). Effect of patient-centered care on patient satisfaction and quality of care. J Nurs Care Qual.

[13] Alessandrini E, Varadarajan K, Alpern ER, Gorelick MH, Shaw K, Ruddy RM (2011). Emergency department quality: an analysis of existing pediatric measures. Acad Emerg Med.

[14] Lindsay P, Schull M, Bronskill S, Anderson G (2002). The development of indicators to measure the quality of clinical care in emergency departments following a modified-delphi approach. Acad Emerg Med.

[15] Weng S-J, Tsai M-C, Tsai Y-T, Gotcher DF, Chen C-H, Liu S-C (2019). Improving the efficiency of an emergency department based on activity-relationship diagram and radio frequency identification technology. Int J Environ Res Public Health.

[16] Wiler JL, Griffey RT, Olsen T (2011). Review of modeling approaches for emergency department patient flow and crowding research. Acad Emerg Med.

[17] Castner J, Suffoletto H (2018). Emergency department crowding and time at the bedside: a wearable technology feasibility study. J Emerg Nurs.

[18] Soremekun OA, Shofer FS, Grasso D, Mills AM, Moore J, Datner EM (2014). The effect of an emergency department dedicated midtrack area on patient flow. Acad Emerg Med.

[19] Walsh A, Bodaghkani E, Asghari S, Etchegary H, Alcock L, Patey C, et al. The impact of patient-centered care in the emergency department: a systematic review. [Internet]. [cited 2021 Jun 15]. Available from: https://www.crd.york.ac.uk/PROSPERO/display_record.php?RecordID=189752 10.1186/s12245-022-00438-0 PMC9367087 35953783

[20] Strategy for patient-oriented research - patient engagement framework - CIHR [Internet]. [cited 2021 May 10]. Available from: https://cihr-irsc.gc.ca/e/48413.html

[21] Forsythe L, Heckert A, Margolis MK, Schrandt S, Frank L (2018). Methods and impact of engagement in research, from theory to practice and back again: early findings from the Patient-Centered Outcomes Research Institute. Qual Life Res.

[22] Pluye P, Gagnon M-P, Griffiths F, Johnson-Lafleur J (2009). A scoring system for appraising mixed methods research, and concomitantly appraising qualitative, quantitative and mixed methods primary studies in mixed studies reviews. Int J Nurs Stud.

[23] 8.4.2 MMSR questions that take a convergent segregated approach to synthesis and integration - JBI Manual for Evidence Synthesis - JBI GLOBAL WIKI [Internet]. [cited 2021 Jun 20]. Available from: https://wiki.jbi.global/display/MANUAL/8.4.2+++MMSR+questions+that+take+a+CONVERGENT+SEGREGATED+approach+to+synthesis+and+integration

[24] Sattar R, Lawton R, Panagioti M, Johnson J (2021). Meta-ethnography in healthcare research: a guide to using a meta-ethnographic approach for literature synthesis. BMC Health Serv Res.

[25] France EF, Cunningham M, Ring N, Uny I, Duncan EAS, Jepson RG (2019). Improving reporting of meta-ethnography: the eMERGe reporting guidance. BMC Med Res Methodol.

[26] Nicholas DB, Muskat B, Zwaigenbaum L, Greenblatt A, Ratnapalan S, Kilmer C, Craig W, Roberts W, Cohen-Silver J, Newton A, Sharon R. Patient- and family-centered care in the Emergency Department for Children with Autism. Pediatrics. 2020;145(Supplement_1):S93–S98. 10.1542/peds.2019-1895L 10.1542/peds.2019-1895L 32238535

[27] Innes K, Elliott D, Plummer V, Jackson D. Emergency department waiting room nurses in practice: an observational study. J Clin Nurs. 2018;27(7-8):e1402–e1411. 10.1111/jocn.14240. 10.1111/jocn.14240 29266573

[28] Polevoi SK, Jewel JS, McCulloch CE, Grimes B, Govindarajan P, Hauswald M (2013). Marked Reduction in Length of Stay for Patients With Psychiatric Emergencies After Implementation of a Comanagement Model. Acad Emerg Med.

[29] Frank C, Asp M, Dahlberg K. Patient participation in emergency care – a phenomenographic study based on patients’ lived experience. Int Emerg Nurs. 2009;17(1):15–22. S1755599X0800116X. 10.1016/j.ienj.2008.09.003. 10.1016/j.ienj.2008.09.003 19135011

[30] Frank C, Arg M, Dahlberg K (2009). Patient participation in emergency care - a phenomenographic analysis of caregivers’ conceptions. J Clin Nurs..

[31] Steven C, Rogers LC, Griffin PD, Masso M, Stevens L, Mangini SR (2015). Smith Cares. Pediatric Emergency Care..

[32] Wilhel K, Finch A, Kotze B, Arnold K, McDonald G, Sternhell P, Hudson B (2007). The Green Card Clinic: Overview of a Brief Patient-Centred Intervention Following Deliberate Self-Harm. Aust Psychiatry..

[33] Walker W, Deacon K. Nurses’ experiences of caring for the suddenly bereaved in adult acute and critical care settings and the provision of person-centred care: a qualitative study. Intensive Crit Care Nurs. 2016;3339–47. S0964339715001172. 10.1016/j.iccn.2015.12.005. 10.1016/j.iccn.2015.12.005 26853502

[34] Trethewey SP, Deepak S, Saad S, Hughes E, Tadros G (2019). Evaluation of the Psychiatric Decisions Unit (PDU): effect on emergency department presentations and psychiatric inpatient admissions. Postgraduate Med J..

[35] Zeller S, Calma N, Stone A (2014). Effect of a Regional Dedicated Psychiatric Emergency Service on Boarding and Hospitalization of Psychiatric Patients in Area Emergency Departments. West J Emerg Med..

[36] Wang L-H, Goopy S, Lin C-C, Barnard A, Han C-Y, Liu H-E (2016). The emergency patient's participation in medical decision-making. J Clin Nurs..

[37] Heifetz M, Lunsky Y. Implementation and evaluation of health passport communication tools in emergency departments. Res Dev Disabil. 2018;7223–32. S089142221730255X. 10.1016/j.ridd.2017.10.010. 10.1016/j.ridd.2017.10.010 29080483

[38] Owens KR. Patient-centered provider behaviors and disclosure of intimate partner violence in a psychiatric emergency setting. Doctoral Dissertation, University of Pittsburgh; 2007.

[39] Robinson N (1991). A patient-centered framework for restructuring care. JONA..

[40] Constand MK, MacDermid JC, Dal Bello-Haas V, Law M (2014). Scoping review of patient-centered care approaches in healthcare. BMC Health Serv Res.

[41] Bamm EL, Rosenbaum P (2008). Family-centered theory: origins, development, barriers, and supports to implementation in rehabilitation medicine. Arch Phys Med Rehabil.

