# Supplementary material for: Patient-centered care in the emergency department: a systematic review and meta-ethnographic synthesis
Source: Int J Emerg Med. 2022 Aug 11;15:36. doi: 10.1186/s12245-022-00438-0 (PMC9367087; doi:10.1186/s12245-022-00438-0)
Supplement: Supplementary file 1 — Additional file 1: Appendix A. Sample search strategy. [file 12245_2022_438_MOESM1_ESM.docx]

# **Appendix A: Sample search strategy**

| **MEDLINE (PubMed) & Cochrane** | |
| --- | --- |
| #1 | "Patient Participation"[Mesh] OR "Patient-Centered Care"[Mesh] |
| #2 | "patient participation" OR "patient engagement" OR "patient centered care" OR "patient centred care" OR "person centered care" OR "person centred care" OR "patient involvement" OR "patient empowerment" OR "patient activation" OR "patient centered" OR "patient centred" OR "patient focused" |
| #3 | #1 OR #2 |
| #4 | (("Emergency Medical Services"[Mesh] OR "Emergency Treatment"[Mesh]) OR ( "Emergency Nursing"[Mesh] OR "Emergency Service, Hospital"[Mesh] OR "Emergency Medicine"[Mesh] OR "Emergency Services, Psychiatric"[Mesh] OR "Pediatric Emergency Medicine"[Mesh] )) OR "Crisis Intervention"[Mesh] |
| #5 | "emergency department" OR "emergency departments" OR "emergency medicine" OR "emergency services" OR "emergency unit" OR "emergency units" OR "emergency ward" or "emergency room" OR "emergency rooms" OR ((psychiatric or mental) AND (emergency or emergencies or crisis)) |
| #6 | #4 OR #5 |
| #7 | "Mental Disorders"[Mesh] |
| #8 | "mental illness" OR "mental health" OR "mental disorder" OR "mental disorders" |
| #9 | #7 OR #8 |
| #10 | #3 AND #6 AND #9 |

| **CINAHL** |  |
| --- | --- |
| #1 | MH "Patient Centered Care" OR MH “Consumer Participation” |
| #2 | "patient participation” OR "patient engagement" OR "patient centered care" OR "patient centred care" OR "person centered care" OR "person centred care" OR "patient involvement" OR "patient empowerment" OR "patient activation" OR "patient centered" OR "patient centred" OR "patient focused" |
| #3 | #1 OR #2 |
| #4 | (MH "Emergency Service") OR (MH "Psychiatric Emergencies") OR (MH "Emergency Services, Psychiatric") OR (MH "Emergency Patients") OR (MH "Emergency Treatment") |
| #5 | "emergency department" OR "emergency departments" OR "emergency medicine"  OR "emergency services" OR "emergency unit" OR "emergency units" OR "emergency ward" or "emergency room" OR "emergency rooms" OR ((psychiatric or mental) AND (emergency or emergencies or crisis)) |
| #6 | #4 OR #5 |
| #7 | (MH "Mental Disorders+") OR (MH "Mental Health") |
| #8 | "mental illness" OR "mental health" OR "mental disorder" OR "mental disorders" |
| #9 | #7 OR #8 |
| #10 | #3 AND #6 AND #9 |
|  |  |
| **PsycInfo** |  |
| #1 | DE "Client Participation" |
| #2 | "patient participation” OR "patient engagement" OR "patient centered care" OR "patient centred care" OR "person centered care" OR "person centred care" OR "patient involvement" OR "patient empowerment" OR "patient activation" OR "patient centered" OR "patient centred" OR "patient focused" |
| #3 | #1 OR #2 |
| #4 | (DE "Emergency  Medicine" OR DE  "Emergency Services"  OR DE "Crisis  Intervention") OR (DE  "Crisis Intervention  Services") |
| #5 | "emergency department" OR "emergency departments" OR "emergency medicine"  OR "emergency services" OR "emergency unit" OR "emergency units" OR "emergency ward" or "emergency room" OR "emergency rooms" OR ((psychiatric or mental) AND (emergency or emergencies or crisis)) |
| #6 | #4 OR #5 |
| #7 | DE "Chronic Mental Illness" OR DE "Mental Health and Illness Assessment" OR DE "Mental Disorders" |
| #8 | "mental illness" OR "mental health" OR "mental disorder" OR "mental disorders" |
| #9 | #7 OR #8 |
| #10 | #3 AND #6 AND #9 |

| **EMBASE** | |
| --- | --- |
| #1 | 'patient care'/exp OR 'patient participation'/exp |
| #2 | "patient participation" OR "patient engagement" OR "patient centered care" OR "patient centred care" OR "person centered care" OR "person centred care" OR "patient involvement" OR "patient empowerment" OR "patient activation" OR "patient centered" OR "patient centred" OR "patient focused" |
| #3 | #1 OR #2 |
| #4 | 'emergency health service'/exp OR 'emergency care'/exp OR 'psychiatric emergency service'/exp OR 'emergency treatment'/exp OR 'pediatric emergency medicine'/exp OR 'crisis intervention'/exp OR 'hospital emergency service'/exp |
| #5 | "emergency department" OR "emergency departments" OR "emergency medicine" OR "emergency services" OR "emergency unit" OR "emergency units" OR "emergency ward" or "emergency room" OR "emergency rooms" OR ((psychiatric or mental) AND (emergency or emergencies or crisis)) |
| #6 | #4 OR #5 |
| #7 | 'mental health'/exp OR 'mental disease'/exp |
| #8 | "mental illness" OR "mental health" OR "mental disorder" OR "mental disorders" |
| #9 | #7 OR #8 |
| #10 | #3 AND #6 AND #9 |
